# Atrial Fibrillation and Non-cardiovascular Diseases: A Systematic Review

**DOI:** 10.5935/abc.20150142

**Published:** 2015-11

**Authors:** Cátia Ferreira, Rui Providência, Maria João Ferreira, Lino Manuel Gonçalves

**Affiliations:** 1 Faculdade de Medicina da Universidade de Coimbra, Coimbra, Portugal; 2 Serviço de Cardiologia – Centro Hospitalar e Universitário de Coimbra, Coimbra, Portugal

**Keywords:** Heart Failure, Atrial Fibrillation / mortality, Arrhythmias / therapeutic use, Neoplasms

## Abstract

Atrial fibrillation (AF) is the most common cardiac arrhythmia and is associated with an unfavorable prognosis, increasing the risk of stroke and death. Although traditionally associated with cardiovascular diseases, there is increasing evidence of high incidence of AF in patients with highly prevalent noncardiovascular diseases, such as cancer, sepsis, chronic obstructive pulmonary disease, obstructive sleep apnea and chronic kidney disease. Therefore, considerable number of patients has been affected by these comorbidities, leading to an increased risk of adverse outcomes.

The authors performed a systematic review of the literature aiming to better elucidate the interaction between these conditions.

Several mechanisms seem to contribute to the concomitant presence of AF and noncardiovascular diseases. Comorbidities, advanced age, autonomic dysfunction, electrolyte disturbance and inflammation are common to these conditions and may predispose to AF.

The treatment of AF in these patients represents a clinical challenge, especially in terms of antithrombotic therapy, since the scores for stratification of thromboembolic risk, such as the CHADS_2_ and CHA_2_DS_2_VASc scores, and the scores for hemorrhagic risk, like the HAS-BLED score have limitations when applied in these conditions.

The evidence in this area is still scarce and further investigations to elucidate aspects like epidemiology, pathogenesis, prevention and treatment of AF in noncardiovascular diseases are still needed.

## Introduction

Atrial fibrillation (AF) is the most common cardiac arrhythmia, occurring in 1.5-2.0% of the general population^1^. The presence of AF is associated with unfavorable prognosis. In addition to be associated with a five-fold higher risk^2^ of stroke and a three-fold incidence of congestive heart failure^1^, FA also contributes to higher mortality. Even in the absence of valvular heart diseases or pre-existing cardiovascular disease, AF doubles the mortality risk in men (multivariate OR, 2.4 [95% CI, 1.8 to 3.3]) and in women (multivariate OR, 2.2 [95% CI, 1.6 to 3.1])^3^, suggesting that AF is a prognostic marker in noncardiovascular diseases.

Although often underestimated, noncardiovascular diseases are closely associated with AF, either as a risk factor for AF development^4^ or as cause of death^5^. The aim of this review was to present the association between AF and noncardiovascular diseases, by describing its underlying mechanisms and its therapeutic and prognostic implications.

## Methods

A systematic review was undertaken using Pubmed database for articles published up to February 2015, using the terms “atrial fibrillation”, combined with some of the noncardiovascular diseases frequently associated with AF supplementary material (MS).

Particular emphasis has been given to more prevalent diseases and those with stronger causal association with mortality in patients with AF. Thus, five conditions were more extensively explored: cancer, sepsis, chronic obstructive pulmonary disease (COPD), obstructive sleep apnea (OSA), and chronic kidney disease (CKD).

## Results

A great variety of conditions are currently associated with AF (Table 1)^4^. Due to the increased mortality caused by FA, not only the risk factors, but also specific causes of death are important to identify. A study on mortality based on the subjects from The Randomized Evaluation of Long-Term Anticoagulant Therapy (RE-LY) trial^5^ verified that the majority of deaths are not related to stroke in anticoagulated atrial fibrillation patients. Although cardiac diseases continue to be the most common causes of death, noncardiovascular deaths accounted for 35.8% of all deaths. In this category, cancer was the most frequent cause of death, followed by respiratory failure (5.7%) and infection (4.45%)^5^.

**Tabela 1:** Fatores de risco associados à fibrilação atrial (adaptado de Kirchhof e cols.^4^)

**Conventional risk factors**
Advanced age
Male gender
Coronary disease
Hypertension (> 140/90 mmHg)
Heart failure
Valvular heart diseases
Diabetes mellitus
Hyperthyroidism
**Less established risk factors**
Chronic obstructive pulmonary disease
Dilation of left atrium
Atrial conduction delay / PR interval
Hypertrophy of left ventricle
Diastolic dysfunction of left ventricle
Obesity
Obstructive sleep apnea
Genetic factors
Arterial pressure / increased pulse pressure
Chronic kidney disease
Inflammation
Increased natriuretic peptides
Excessive resistance exercise
Excessive alcohol consumption
Height

Therefore, when assessing less established risk factors for AF, several noncardiovascular diseases are identified, notably cancer, sepsis, COPD, OSA and CKD. Since the number of patients suffering from these conditions is limited in large-scale studies, most of data in the following sections have been collected from epidemiological records and studies.

### Cancer

Although cancer has been recently associated with AF, there are few studies confirming this association. Guzzetti et al^6^ one of the first groups to investigate such association, reported that FA was present in 3.6% of colorectal cancer (CRC) patients or breast cancer patients and in 1.6% of controls, corresponding to at least two times higher likelihood of having AF in patients with cancer (p < 0.01)^6^.

In a cohort study^7^, the prevalence of AF at the moment of cancer diagnosis (2.4%) and the percentage of patients who developed AF after cancer diagnosis (1.8%)^7^ were determined in 24,125 recently diagnosed patients. Erichsen et al^8^, in a case-control study, observed that in patients with AF, 0.59% had a CRC diagnosis within 90 days before their AF diagnosis, compared with only 0.05% of controls (adjusted OR = 11.8; 95% CI 9.3-14.9).

The most common and most studied type of AF is the postoperative AF. Thoracic surgery, especially pulmonary resection for lung cancer, is associated with a significant risk of AF, with variable incidence (Table S-1). According to the Society of Thoracic Surgeons database, 12.6% of 13,906 patients who underwent surgery for lung cancer developed AF after the surgery^9^. On the other hand, the prevalence of postoperative AF in patients who underwent elective surgery for CRC was 4.4%^10^.

In addition, AF may also complicate the course of cancer disease as an adverse drug reaction by several mechanisms including cardiotoxicity (MS).

AF may represent a comorbidity in cancer, since both conditions share several factors predisposing to AF such as advanced age, electrolyte abnormalities, hypoxia, and metabolic disorders. Changes in autonomic nervous system due to the increased sympathetic stimulation by pain or other forms of physical or emotional stress may predispose to AF^11^. In addition, cancer is often associated with a hypercoagulability state and increased thromboembolic risk, which may lead to pulmonary microembolism and AF^6^. AF may also result from an abnormal production of hormone-like peptides and paraneoplastic conditions, including hyperthyroidism and immune reaction against atrial structures^11^.

Inflammation plays an important role in carcinogenesis^12^ and AF may represent an inflammatory complication of cancer (MS)^13^.

AF may also be a direct manifestation of primary neoplasms, metastatic cardiac tumors or tumors of adjacent tissues, such as the lungs and esophagus that invade the heart^11^.

AF has a negative impact on prognosis. Patients who developed AF after surgery for lung cancer experienced higher postoperative mortality as compared with patients without AF (6.7% versus 1.0%, p = 0.024) during hospitalization and intensive care unit (ICU) admissions. AF was also associated with higher long-term mortality among patients alive at 5 years from surgery (HR 3.75, 95% CI 1.44-9.08, p = 0.007)^14^. In patients who underwent surgery for CRC, AF seems also to indicate worse survival^15^.

AF is also associated with two-fold increased risk of thromboembolism and six-fold increased risk of heart failure, even after adjusting for well-known risk factors (adjusted HR 1.98, 95% CI 1.6-2.46, p < 0.001 and 6.3, 95% CI 4.83-8.17, p < 0.001, respectively)^7^.

These findings suggest that both treatment and prevention of AF may be important in cancer patients. However, the treatment of AF in these patients constitutes a challenge, especially in choosing the antithrombotic therapy. Cancer, *per se*, promotes a prothrombotic state, and increases the risk of thromboembolic events in patients with AF. On the other hand, some neoplasms are associated with increased risk of hemorrhage. Also, therapy with warfarin may be problematic in cancer patients due to the concomitant medication or metabolic disorders secondary to cancer, leading to an unpredictable anticoagulant response^11^.

Finally, there are no specific recommendations for AF treatment in patients with neoplasms^16^. The scores for thromboembolic risk prediction, CHADS_2_ or CHA_2_DS_2_VAS_C_, do not include cancer as a variable and may not be appropriate for these patients. An epidemiological study concluded that the CHADS_2_ score may be predictive for thromboembolic risk in patients with AF at the moment of cancer diagnosis, but not among those who developed AF after the diagnosis^7^.

Low-molecular-weight heparin (LMWH) may have an antineoplastic potential and positively influences cancer patients survival, representing a more appropriate alternative than coumarins^17^. Dalteparin has been associated with a better survival in patients with solid tumors without metastatic diseases and venous thromboembolic events as compared with coumarin derivatives^18^. In line with this evidence, the American College of Chest Physicians recommends the use of LMWH instead of warfarin in patients with cancer and thromboembolic disease in the first 3-6 months of antithrombotic therapy^19^. However, the long-term effect of LMHW on cancer patients is still unknown^11^.

Several studies have identified AF following thoracic surgery for lung cancer (Table 2). In this context, the brain natriuretic peptide (BNP) has been investigated as a predictive marker of postoperative AF. Both increased preoperative and postoperative values are strong independent predictors of AF (RR 27.9, 95% CI 13.2-58.9, p < 0.001, and RR20.1 95% CI 5.8-69.4, p < 0.001, respectively)^20^. Salvatici et al. identified a cut-off point of 182 ng/L as a predictive marker of postoperative AF^21^. However, a cut-off point of 30 pg/mL has a 93% specificity to predict AF after thoracic surgery for lung cancer^22^. Echocardiographic indexes may also be useful, especially if they indicate diastolic dysfunction of left ventricle^23^.

**Tabela 2:** Preditores de fibrilação atrial após resseção pulmonar por neoplasia maligna^9,14,64^

Atrial fibrillation predictors after pulmonary resection for malignant neoplasm
Advanced age
Male gender
Prolonged surgery
Advanced cancer staging
Surgical complications
Postoperative blood transfusion requirement
History of hypertension and preoperative paroxysmal atrial fibrillation
Elevated brain natriuretic peptide levels in the preoperative and postoperative periods
Echocardiographic indexes of diastolic dysfunction of left ventricle

Some drugs have been studied for the prevention and treatment of postoperative AF (MS).

### Sepsis

New-onset AF is a complication commonly seen in ICUs, drawing more and more attention due to its frequency and impact on patient’s prognosis (Table S-2). In the ICUs, AF is particularly common in patients with sepsis, which has been identified as an independent predictor of AF in ICU of cardiac patients (OR 6.5, 95% CI 2.0-21.1, p = 0.002)^24^, or surgical patients^25^. In a systematic review, the weighted mean incidence of new-onset AF was 8% (0-14%), 10% (4-23%) and 23% (6-46%) in patients with sepsis, severe sepsis and septic shock, respectively^26^.

Sepsis is characterized by a systemic release of proinflammatory cytokines, increased levels of circulating catecholamines, electrolyte disturbances, autonomic dysfunction, and may be complicated by organic dysfunction^27^. Changes in intravascular volume and cardiovascular compromise frequently lead to hypotension and elevated lactate level^24,28^. However, risk factors for AF in general population, including advanced age, male gender, Caucasian race, heart failure, and obesity have been associated with AF development in sepsis^26^. All these characteristics may cause AF in sepsis, although increasing evidence has supported that systemic inflammatory response, *per se*, is the main contributing factor to AF, with increased serum C-reactive protein (CRP) before the onset of AF^24^.

New-onset AF in patients with sepsis has been associated with longer stay in the ICU and increased risk for ischemic stroke (adjusted OR 2.70, 95% CI 2.05 to 3.57, p < 0.001)^26^. Most studies have reported increased acute (ICU or in-hospital) mortality, with estimated ORs varying from 1.07 (95% CI 1.04 to 1.11) and 3.28 (95% CI 1.13 to 9.57) for 28-day mortality^29^. Besides, the development of AF during sepsis may have implications after discharge, since a greater risk of hospitalization for heart failure (HR 1.25; 95% CI, 1.16-1.34), ischemic stroke (HR 1.22; 95% CI, 1.10-1.36), and death (HR 1.04; 95% CI,1.01-1.07) has been observed in the following 5 years^30^.

Treatment of AF in critically ill patients poses a clinical challenge, with no specific recommendations in the literature. An important question to be discussed is whether the association between AF and stroke may lead to an intervention aimed at preventing such complication, such as the cardioversion, anticoagulation, or both. However, it is difficult to maintain sinus rhythm after cardioversion as sepsis persists, additionally to the fact that the damage may be a result of an indiscriminate use of anticoagulants due to coagulation disorders by patients with sepsis, and invasive procedures to which they are exposed^31^. In addition, failure to restore sinus rhythm is associated with increased ICU mortality (71% versus 21%, p = 0.015)^32^.

Therefore, a prophylactic therapy to prevent this complication may be effective, unless patients in higher risk of developing AF during sepsis are appropriately identified (MS)^26^.

### Chronic Obstructive Pulmonary Disease

COPD is an independent risk factor for arrhythmias, especially AF, and cardiovascular morbidity and mortality^13,33^. In a large-scale, retrospective, case-control study, patients with COPD had a 4.41 times higher risk of AF (95% CI 4.00-4.87)^34^ and COPD is present in 10-15% of patients with AF^33^. Decreased pulmonary function is an independent risk factor of AF^35^.

Numerous pathologic processes including concomitant diseases, age, hypoxia, hypercapnia, acidosis, inflammation, electrolyte disturbances, autonomic dysfunction, and pulmonary hypertension may precipitate new-onset or recurrent AF^36^. Right atrial electromechanical delay and the duration of atrial depolarization are significantly prolonged, and propagation of depolarization is inhomogeneous in patients with COPD. These may be the mechanisms underlying the development of AF in COPD patients^37^.

Agents used to improve pulmonary function, notably beta-adrenergic agonists and theophyllines can cause tachyarrhythmias^33^. Agents used in the control of AF, particularly sotalol, propafenone, and non-selective β-blockers, may cause bronchospasm^33^. Pulmonary symptoms in COPD may become worse with AF development, due to excessive, irregular heart rate, as well as reduced diastolic filling of the ventricles^38^.

Therefore, AF and COPD frequently coexist and interact. COPD is an independent predictor of AF progression from paroxysmal to persistent AF (OR 1.51, 95% CI 0.95–2.39, p = 0.088), and is one of the five variables included in the HATCH score, which estimates the probability of this AF progression^39^.

AF in patients with COPD has a negative impact on prognosis. In a large-scale, retrospective study, a 1.98-fold greater risk of hospitalization in patients with AF was observed (95% CI 1.73–2.25)^34^. AF has been also considered an independent mortality factor in exacerbations of COPD (OR 2.66, 95% CI 1.39-5.09, p = 0.003)^40^.

In contrast to neoplasms and sepsis, pulmonary diseases are included in current recommendations (MS). However, there is no specific recommendations regarding antithrombotic therapy^16^. There is a significant risk of thromboembolic events in COPD exacerbations^41^. The last edition of the Global strategy for the diagnosis, management, and prevention of chronic obstructive pulmonary disease (GOLD guidelines)^42^ suggests that thromboprophylactic measures in COPD exacerbations, including the use of subcutaneous heparin or LMWH^42^.

Catheter ablation may be an effective and safe approach to patients with COPD, although may be associated with increased recurrence rate after ablation (OR 1.9, 95% CI 1.07–3.557, p = 0.029)^43^.

### Obstructive Sleep Apnea

OSA is a common respiratory sleep disorder affecting approximately 10% of population^44^, and is associated with cardiac mortality and morbidity. The Sleep Heart Health Study reported a 4-fold higher prevalence of AF in OSA patients (OR 4.02, 95% CI 1.03–15.74)^45^. The risk of AF increases with the severity of OSA^46^. In addition, OSA is more prevalent among patients with AF than in general population. A prospective study reported a strong association between these two conditions (adjusted OR 2.19, 95% CI 1.40-3.42, p = 0.0006)^47^.

AF and OSA share several factors and comorbidities, including advanced age, obesity, hypertension, heart failure and heart disease. OSA is also associated with intermittent hypoxia, acidosis, autonomic disorder, oxidative stress and endothelial dysfunction that may be involved in AF pathophysiology. Additionally, OSA increases inflammatory marker levels, such as CRP, interleukin 6 (IL-6), and tumor necrosis factor-alpha (TNF-α), leading to a proinflammatory state. Obstructive events in OSA cause a negative intrathoracic pressure, contributing to enlargement of atrial chamber, atrial fibrosis and remodeling of pulmonary vessels, which are well-established risk factors of AF^46^.

Few studies have investigated the impact of AF on OSA prognosis. OSA is associated with increased risk of stroke^48^. However, it is unclear whether AF increases the risk of stroke in patients with OSA (MS).

Yaranov et al^49^, in a retrospective study on 5,138 patients, investigated the impact of OSA on stroke rate in patients with AF, and concluded that ischemic stroke was more frequent in patients with OSA compared with patients without (25.4% versus 8.2%, p = 0.006). Even after controlling for age, male gender, and coronary heart disease, the association between OSA and stroke remained significant, indicating that OSA is an independent risk factor for stroke in patients with AF (adjusted odds ratio of 3.65, 95% CI 1.252 to 10.623)^49^. Thus, it becomes relevant to verify whether OSA adds predictive value to the CHA_2_DS_2_VAS_C _score. The risk of stroke in patients with OSA was 1.62 time higher (95% CI 1.155-2.259) in patients with scores of 0, although the presence of OSA in patients with higher scores did not increase the incidence of stroke. Large-scale, prospective studies are needed to determine the role of OSA on thromboembolic risk in patients with AF^49^.

With respect to AF treatment, the presence of OSA significantly reduces the efficacy of pharmacological and nonpharmacological therapies for AF^46^. Current recommendations suggest that sleep study may be considered when OSA in patients with AF is suspected^16^. In addition, there is a strong possibility that treatment with continuous positive airway pressure (CPAP) may have beneficial effects on AF prevention, since it reduces or eliminates many of the mechanisms assumed to associate OSA with AF, markedly hypoxemia, inflammation, sympathetic hyperactivity and hypertension. Also, treatment with CPAP is associated with a lower risk of AF recurrence after cardioversion and ablation^46^.

### Chronic Kidney Disease

Patients with CKD are more likely to develop AF (Table S-5). In The Atherosclerosis Risk in Communities (ARIC) Study, in a cohort of 10,328 individuals with CKD, the incidence of AF was 7.6% during a median follow-up of 10.1 years. The incidence of AF increases as renal function decreases^50^. In addition, CKD is found in nearly 10-15% of AF patients^33^, and AF is associated with increased risk of developing CKD (HR 1.77, 95%CI 1.5-2.1, p < 0.001)^51^.

Regardless of its cause, CKD coexists with a proinflammatory state, which may be implicated in the development of AF. Plasma levels of CRP and IL-6 are elevated in patients with CKD^52^. Another mechanism proposed is that pathological activation of the renin-angiotensin-aldosterone system may lead to atrial fibrosis and atrial remodeling, creating a substrate for the development of AF^50^, including the autonomic dysfunction, found in early stages of CKD^53^. In addition, hemodialysis therapy induces an increase in P-wave duration, which may favor AF onset^54^.

Finally, advanced age and white race are independent predictors of AF in CKD^55^, and cardiovascular comorbidities frequently associated with CKD are risk factors for the development of AF (MS)^50^.

Concomitant presence of AF and CKD is associated with a bad prognosis. AF is associated with a 67% increase in the incidence of end-stage renal disease (ESRD) in patients with CKD (HR 1.67, 95% CI 1.46-1.91)^56^. In a meta-analysis including 19 studies, the presence of CKD in patients with AF increased the thromboembolic risk (HR 1.46, 95% CI 1.20-1.76, p = 0.0001), particularly in CKD (HR 1.83, p 95% CI 1.56-2.14, p < 0.00001)^57^.

AF is also associated with increased mortality, with a 66% increase in relative risk of death at stages 3-5 of CKD (adjusted HR 1.66, 95% CI 1.57-1.77)^56^.

Treatment of AF in CKD consists in a clinical challenge. These patients experience not only higher rates of thromboembolic complications, but also increased hemorrhagic risk, which is exacerbated by warfarin, aspirin, or both^58^. However, when the benefit of anticoagulation is contrasted with the risk of hemorrhage, the risk-benefit ratio tends to favor anticoagulation^59^.

In a meta-analysis, the use of warfarin decreased the incidence of thromboembolic events in CKD patients without ESRD (HR 0.39, 95% CI 0.18-0.86, p < 0.00001)^57^. Recent data on new anticoagulants have suggested similar efficacy and greater safety compared with warfarin^60^, and their promising role in CKD.

Current guidelines recommend anticoagulation with warfarin (INR, international normalized ratio 2-3) in patients with nonvalvular AF and CHA2DS2-VASc score ≥ 2, despite recognizing that anticoagulation increases the hemorrhagic risk in this population (MS)^16^.

## Discussion

AF is commonly associated with other noncardiovascular diseases that affect a great number of patients, including cancer, sepsis, COPD, OSA and CKD. Since AF has an adverse prognosis, understanding how these conditions interact and the more appropriate therapies is essential.

**Figure 1:**
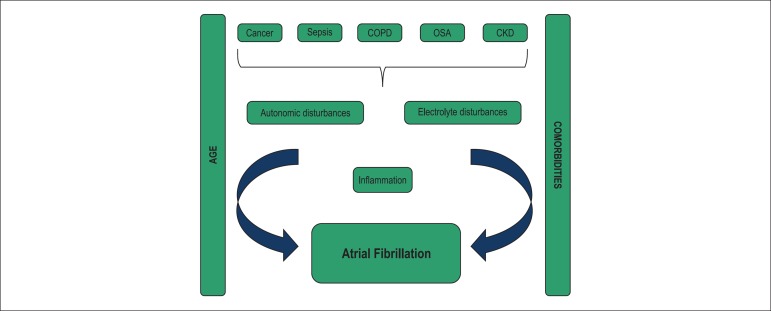
Common mechanisms of atrial fibrillation development. COPD: Chronic obstructive pulmonary disease, OSA: Obstructive sleep apnea, CKD: Chronic kidney disease

All these conditions and AF share well established risk factors, including cardiovascular comorbidities and advanced age. Additionally, they are all associated with autonomic dysfunction, electrolyte and inflammatory disturbances (Figure 1 and Figure S-1).

Inflammation is a common denominator of all conditions, and maybe one of the most important. First, a case-control study has reported a significant increase in CRP in patients with AF, both in patients with structural heart disease and patients with isolated AF^61^. Then, in a population-based study, 5,806 subjects were followed up for a mean of 7.8 years, showed that elevated CRP levels were associated with higher prevalence of pre-existing AF (OR 1.8, 95% CI 1.2-2.5, p = 0.002) and higher risk for developing future AF (OR 1.31, 95% CI 1.08-1.58, p = 0.005)^62^. These studies suggest that systemic inflammatory states, of which CRP is a marker, may induce atrium structural or electrical remodeling, and promote and maintain AF^61,62^. In addition to CRP, the increase in other inflammatory markers’ levels, such as TNF-α, IL-2, IL-6 and IL-8 have been also associated with AF^63^.

The combination of AF with these conditions constitutes a therapeutic challenge. Traditionally, anticoagulant therapy in patients with nonvalvular AF is initiated based on stratification of thromboembolic risk using the CHADS_2_ and CHA_2_DS_2_VASc scores, and hemorrhagic risk using the HAS-BLED score. However, these scoring systems have limitations. Both OSA and CKD are independent risks for stroke in patients with AF, and are not included in the thromboembolic scores. In addition, cancer, *per se*, is associated with increased thromboembolic risk. The hemorrhagic score includes renal function only, although hemorrhagic risk is elevated in some cancers and sepsis, and may not be negligible. Therefore, further studies to validate these and other risk stratification tools in these conditions are needed. Similarly, there are no large-scale studies comparing heart rate with cardiac rhythm, catheter ablation and antithrombotic therapy.

The identification of AF predictors in different pathologies may lead to adoption of prophylactic measures. Although independent risk factors as well as laboratory and echocardiographic markers have been identified in all conditions, their validation in larger samples is still needed for their clinical application.

## Conclusion

The presence of AF in noncardiovascular diseases seems to directly affect their prognosis, and its treatment is still a challenge. Researches in some of these areas are still in initial phase, and further investigations to elucidate aspects like the epidemiology, pathogenesis, prevention and treatment of AF in noncardiovascular diseases are still needed.

Therefore, the diagnosis of a new-onset AF in patients with certain clinical characteristics may justify the screening of some of the diseases previously described. For example, a 50-year old patient who has a strong family history of cancer and develops a new-onset AF in the absence of cardiac disease may justify a cancer screening. Similarly, an obese patient with AF may justify the screening of OSA.
