# Supplementary material for: Atrial Fibrillation and Non-cardiovascular Diseases: A Systematic Review
Source: Arq Bras Cardiol. 2015 Nov;105(5):519–26. doi: 10.5935/abc.20150142 (PMC4651411; doi:10.5935/abc.20150142)
Supplement: Supplementary file 1 [file abc-105-05-0519-suppl01.pdf]

Supplementary Method

We used the terms *"Cancer"*, *"neoplasms"* and *"paraneoplastic"* to identify potentially relevant studies for sepsis; *"chronic obstructive pulmonary disease"*, *"COPD"* and *"chronic bronchitis"* for COPD; *"sleep apnea"*, *"obstructive sleep apnea"* and *"sleep-disordered breathing"* for OSA; and *"chronic kidney disease"* and *"renal disease"* for CKD.

Coronary heart disease, hypertension, heart failure and valvular heart disease were not included as they were classified as cardiovascular diseases. Other noncardiovascular diseases, namely hyperthyroidism and diabetes mellitus, although associated with AF, were similarly excluded as they were considered conventional risk factors,[1] and thus their association with AF has already been explored in previous studies. Obesity, however, although consistently emerges as a risk factor for AF, was not included due to its intrinsic connection with OSA.

Bibliographic references of relevant articles consisted of journals and articles associated. The search was not limited to a unique publication, language or specific quality criteria.

Suplemmentary Results

Cancer

Pathophysiology

AF may act as an adverse drug reaction and complicate the course of cancer patients. AF may be induced by various cytostatics, such as Anthracyclines (Doxorubicin, Mitoxantrone), Ifosfamide, Gemcitabine, Melphalan, Cisplatin, Docetaxel, 5-Fluorouracil and Etoposide, high doses of Corticosteroids, Bisphosphonates, antiemetic agents like Ondansetron, and targeted therapies, by several mechanisms, including cytotoxicity[2–8].

AF may also represent an inflammatory complication of cancer. Indeed, AF was more commonly observed in patients with elevated postoperative neutrophil counts (OR 3.2, 95% CI 1.3-7.8, $p = 0.01$), and after open versus laparoscopic colectomy (OR 3.3, 95% CI 1.3-8, $p = 0.008$), suggesting that open colectomy causes a more severe systemic inflammatory response[9].

Prophylaxis

Administration of 300 mg of amiodarone intravenously over 20 minutes immediately after surgery for lung cancer and an oral dose of 600mg twice daily during the first five postoperative days reduced the risk of AF by 23%[10]. Nojiri et al[11] reported that patients with elevated BNP levels (> 30 pg/mL) who received low-dose human atrial natriuretic peptide had lower incidence of postoperative AF than patients who received placebo.

Treatment

Landiolol, an ultra-short-acting beta-blocker, when administered to a small group of patients who developed AF after lung resection, experienced a significant reduction in heart rate and early restoration of sinus rhythm as compared to verapamil and digoxin[12].

Sepsis

Prophylaxis

A recent study investigated the effect of esmolol in patients with septic shock[13]. Although reduction in heart rate may lead to improvement of cardiovascular function, treatment of sinus tachycardia, and consequently potentially prevent AF, the use of esmolol in sepsis is still controversial. Further studies to establish the recommendations regarding prophylaxis are needed[14].

COPD

Treatment

According to current recommendations, reversal of hypoxemia and acidosis should be the first therapeutic approaches in new-onset AF. However, in patients who become hemodynamically unstable, synchronized cardioversion should be considered, although the strategy for rhythm control may be ineffective until the respiratory decompensation is corrected.

For ventricular rate control, diltiazem or verapamil are the recommended drugs for COPD patients. The use of β-adrenergic agonists and theophylline is discouraged, since they may precipitate atrial fibrillation and make ventricular rate control difficult. Non-selective beta-blockers, sotalol, propafenone and adenosine are contraindicated in patients with bronchospasm[15].

Obstructive Sleep Apnea

Prognosis

Few studies have investigated the effect of AF on OSA. OSA is associated with increased risk of stroke, but it is not clear whether AF increases the risk of stroke in OSA. The Sleep Heart Health study[16], a prospective study which followed up 5,422 individuals with no history of stroke for a mean of 8.7 years, reported that OSA increases the risk of stroke, particularly in men in the highest severity quartile (obstructive apnea-hypopnea index > 19: adjusted HR 2.86, 95% CI 1.1-7.4). After secondary analyses that excluded individuals with AF, lower OR for stroke was observed in OSA patients, with no change in overall results, suggesting that AF does not fully explain the association between OSA and AF. However, the proportion of patients with AF in this study was small (2%), and the authors suggested that underdiagnosed paroxysmal AF was a mediating factor.

More recently, a case-control study involving 108 individuals reported a significant association between AF and stroke, even after adjusting for other risk factors (corrected OR 5.34, 95% CI 1.79-17.29)[17]. Further studies to confirm whether AF increases the risk of stroke in patients with OSA are necessary.

Chronic Kidney Disease

Pathophysiology

In addition to cardiovascular diseases, other comorbidities are commonly encountered in AF and CKD. Curiously, the combination of CKD and anemia increases substantially the risk of stroke (HR 5.43, 95% CI 2.04-14.41)[18], which may be related to an increased risk for AF. In fact, anemia, a common complication of CKD, and CKD are independent risk factors of AF, and a recent study demonstrated a synergic association between CKD and anemia for AF onset[19].

Treatment

Several international anticoagulation therapy guidelines in CKD are currently available. In 2011, the Kidney Disease Outcomes Quality Initiative recommended that anticoagulation therapy should only be prescribed for patients with CKD as a secondary prevention of stroke and careful monitoring of patients, and not as primary prevention, since these patients were not included in controlled, randomized studies[20]. However, the 2014 AHA/ACC/HRS guidelines support the prescription of warfarin (INR 2.0-3.0) for oral anticoagulation for patients with nonvalvular AF, $CHA_2DS_2VAS_C \geq 2$, and who have ESRD or are on hemodialysis, recognizing that anticoagulation increases the hemorrhagic risk in this population. With respect to AF in moderate to severe CKD with CHA2DS2-VASc ≥ 2, treatment with lower doses of direct thrombin or factor Xa inhibitors may be considered, although safety and efficacy have not been established[15].

Supplementary table 1 – Atrial fibrillation and cancer

Author, reference	Study design	Population (patients)	Main results
Guzzetti et al[21], 2002	Retrospective unicenter	1,463	Prevalence of AF: 5% in patients with CRC *vs* 2% in controls
Guzzetti et al[22], 2008	Retrospective unicenter	2,339	Prevalence of AF: 3.6% in patients with CRC and breast cancer *vs* 1.6% in controls
Hu et al[23], 2013	Retrospective population-based	24,125	Prevalence of AF at cancer diagnosis: 2.4%. New-onset AF: 1.8%
Erichsen et al[24], 2012	Retrospective population-based	Cases: 28,333<br>Controls: 283,260	Diagnosis of CRC: 0.59% in patients with AF *vs* 0.05% without AF
Dyszkiewicz et al[25], 1998	Retrospective unicenter	298	Prevalence of FA after pulmonary resection for lung cancer: 8.4%
Roselli et al[26], 2005	Retrospective unicenter	604	Prevalence of FA after pulmonary resection for lung cancer: 19%
Salvatici et al[27], 2010	Prospective unicenter	400	Prevalence of AF after pulmonary resection for lung cancer: 18%
Nojiri et al[28], 2010	Prospective unicenter	126	Prevalence of AF after pulmonary resection for lung cancer: 23%
Imperatori et al[29], 2012	Prospective unicenter	454	Prevalence of AF after pulmonary resection for lung cancer: 9.9%
Murthy et al[30], 2003	Retrospective unicenter	921	Prevalence of AF after esophagectomy: 22%
Nojiri et al[11], 2012	Prospective unicenter	40	Prevalence of AF after pulmonary resection for lung cancer: 60%
Onaitis et al[31], 2010	Retrospective using The Society of Thoracic Surgeons database	13,906	Prevalence of AF after pulmonary resection for lung cancer: 12.6%
Siu et al[9], 2005	Retrospective unicenter	563	Prevalence of AF after colectomy for CRC: 4.4%
**Prophylaxis and treament**			
Riber et al[10], 2012	Prospective unicenter, double-blind, randomized, controlled study	254	Postoperative amiodarone as prophylaxis: reduced the risk of AF from 32% to 9%
Nojiri et al[11], 2012	Prospective unicenter, double-blind, randomized, controlled study	40	Prophylaxis with atrial natriuretic peptide: reduced the postoperative AF (10% *vs* 60%)
Nojiri et al[12], 2011	Prospective unicenter	30	Landiolol *vs* verapamil+digoxin: time to cardioversion was shorter in the landiolol group (8.1 vs 23 horas)

AF: Atrial fibrillation; CRC: Colorectal cancer.

Supplementary Material

Supplementary table 2 – Atrial fibrillation and sepsis

Author, reference	Study design	Population (patients)	Main results
Kuipers et al[14], 2014	Systematic review and meta-analysis	460,096	– Incidence of AF: 8% (0-14%) in sepsis, 10% (4-23%) in severe sepsis and 23% (6-46%) in septic shock<br>– Increased mortality in acute phase with estimated adjusted OR between 1.07 e 3.28
Christian et al[32] 2008	Retrospective unicenter	274	– Incidence of AF: 3% in severe sepsis and 11% in septic shock<br>– ICU mortality: 69% in patients with AF *vs* 40% without AF<br>– Increased hospital stay length in patients with AF
Salman et al[33], 2008	Retrospective unicenter	81	– Incidence of AF: 14% in sepsis, 23% in severe sepsis and 37% in septic shock<br>– Increased mortality in the first 28 days: 72% in patients with AF *vs* 38% without AF
Meierhenrich et al[34], 2010	Prospective unicenter	629	- Incidence of AF in septic shock: 46%<br>– No statistically significant increase in ICU mortality was observed (44% with AF *vs* 33% without AF)
Lee-Iannotti et al[35], 2012	Retrospective, population-based	3,144,787	– Incidence of AF: 5.9% in severe sepsis *vs* 0.65% without severe sepsis<br>– Risk of in-hospital stroke: 2.6% in severe sepsis and new-onset AF *vs* 0.6% in severe sepsis without AF
Walkey et al[36], 2014	Retrospective using The Medicare 5% database	138,722	New-onset AF during sepsis was associated with increased 5-year of risk of hospitalization for heart failure (11.2% *vs* 8.2%), ischemic stroke (5.3% vs 4.7%) and death (74.8% *vs* 72.1%)
**Profilaxia**			
Morelli et a[13], 2013	Open-label, randomized phase 2, unicenter	154	Esmolol: reduction of heart rate in the first 96 hours (-28/min *vs* -6/min)

AF: Atrial fibrillation; ICU: Intensive care unit.

Supplementary table 3 – Atrial fibrillation and chronic obstructive pulmonary disease

Autor, referência	Desenho do estudo	Population (patients)	Main results
Sidney et al[37], 2005	Retrospective multicentric	91,932	– Prevalence of AF: 4.7% in patients with COPD *vs* 1.1% without COPD<br>– A 1.98 fold-greater risk of hospitalization in COPD and AF *vs* COPD without AF
Buch et al[38], 2003	Prospective multicentric	13,460	Reduction in pulmonary function is an independent predictor of AF
Li et al[39], 2014	Prospective population-based	15,004	– The incidence of AF inversely correlates with FEV1<br>– Moderate/severe airflow obstruction associates with AF incidence
Steer et al[40], 2012	Prospective multicentric	920	AF is an independent predictor of mortality in COPD exacerbations
Fuso et al[41], 1995	Retrospective unicentric	590	AF is an independent predictor of mortality in COPD exacerbations

FEV1: Forced expiratory volume in 1 second; AF: Atrial fibrillation; COPD: Chronic obstructive pulmonary disease.

Supplementary table 4 – Atrial fibrillation and obstructive sleep apnea

Author, reference	Study design	Population (patients)	Main results
Mehra et al[42], 2006	Retrospective multicentric	3,295	– Prevalence of AF: 4.8% in patients with sleep disorders *vs* 0.9% without sleep disorders<br>– Patients with sleep disorders have 4-fold greater risk of AF
Gami et al[43], 2007	Retrospective unicentric	3,542	The magnitude of nocturnal oxygen desaturation is an independent risk factor for AF in patients aged less than 65 years.
Gami et al[44], 2004	Prospective unicentric	524	Prevalence of OSA: 49% in patients with AF *vs* 32% without AF
Redline et al[16], 2010	Prospective multicentric	5,422	– Increased risk for stroke in patients with OSA, particularly in men with moderate/severe OSA<br>– AF does not explain the association between stroke and OSA
Mansukhani et al[17], 2013	Retrospective multicentric	108	– Patients with OSA and stroke have a higher incidence of AF
Yaranov et al[45], 2015	Retrospective multicentric	5,138	– Stroke in patients with AF: 25.4% in patients with OSA *vs* 8.2% without OSA<br>– OSA is an independent factor for stroke in patients with AF

AF: Atrial fibrillation; OSA: Obstructive sleep apnea.

Tabela Suplementar 5 – Atrial fibrillation and chronic kidney disease

Author, reference	Study design	Population (patients)	Main results
Ananthapanyasut et al[46], 2010	Retrospective multicentric	1,010	Prevalence of AF in nondialysis patients with CKD: 21.2%
Soliman et al[47], 2010	Prospective multicentric	3,267	Prevalence of AF in CKD (but not ESRD) patients: 18%
Bansal et al[48], 2014	Prospective multicentric	81,088	– Prevalence of AF in CKD (stage 3-5): 7.7%<br>– AF is independently associated with an increased risk of death in adults with CKD
Alonso et al[49], 2011	Prospective population-based	10,328	Reduction in renal function and albuminuria have a strong association with the incidence of AF
Nelson et al[50], 2012	Retrospective using the Medicare 5% database	1,092,649	– CKD, particularly 3-5 stage CKD, is associated with increased risk of AF.<br>– After AF development, the mortality rate is higher in patients with advanced CKD than in patients without CKD
Watanabe et al[51], 2009	Prospective multicentric	235,818	– AF increases the risk of CKD development
Bansal et al[52], 2013	Prospective multicentric	206,229	– AF was independently associated with an increased risk of ESRD in patients with CKD
Providência et al[53], 2014	Systematic review and meta-analysis	379,506	– The presence of CKD in patients with AF increases the thromboembolic risk, particularly in patients with ESRD<br>– Warfarin reduced the incidence of thromboembolic events in patients with non-end-stage CKD

AF: Atrial fibrillation; CKD: Chronic kidney disease; ESRD: End-stage renal disease.

Supplementary Material

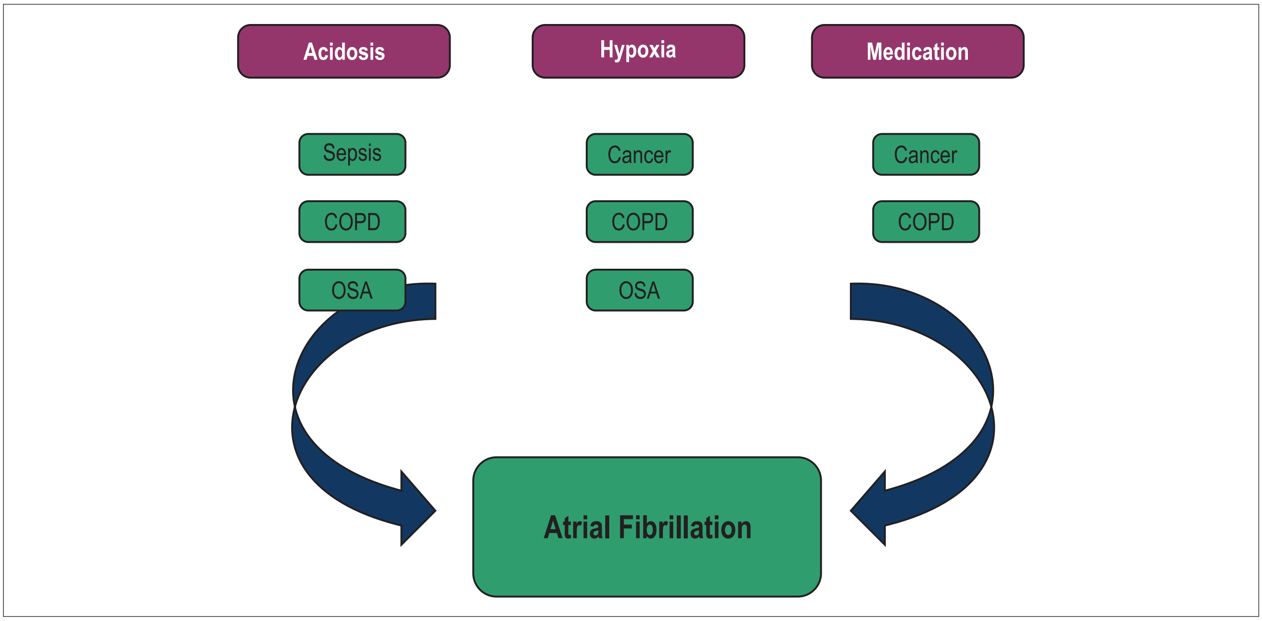

Supplementary figure 1 – *Common mechanisms in some diseases associated with development of atrial fibrillation. COPD: Chronic obstructive pulmonary disease; OSA: Obstructive sleep apnea.*

References

1. Kirchhof P, Lip GY, Van Gelder IC, Bax J, Hylek E, Kaab S, et al. Comprehensive risk reduction in patients with atrial fibrillation: emerging diagnostic and therapeutic options - a report from the 3rd Atrial Fibrillation Competence NETwork/European Heart Rhythm Association consensus conference. Europace. 2012;14(1):8-27.
2. van der Hooft CS, Heeringa J, Brusselle GG, Hofman A, Witteman JC, Kingma JH, et al. Corticosteroids and the risk of atrial fibrillation. Arch Intern Med. 2006;166(9):1016-20.
3. Christiansen CF, Christensen S, Mehnert F, Cummings SR, Chapurlat RD, Sørensen HT. Glucocorticoid use and risk of atrial fibrillation or flutter: a population-based, case-control study. Arch Intern Med. 2009;169(18):1677-83.
4. Velagapudi P, Turagam MK, Kocheril AG. Atrial fibrillation in cancer patients: an underrecognized condition. South Med J. 2011;104(9):667-8.
5. Erichsen R, Christiansen CF, Frøslev T, Jacobsen J, Sørensen HT. Intravenous bisphosphonate therapy and atrial fibrillation/flutter risk in cancer patients: a nationwide cohort study. Br J Cancer. 2011;105(7):881-3.
6. Khouri MG, Douglas PS, Mackey JR, Martin M, Scott JM, Scherrer-Crosbie M, et al. Cancer therapy-induced cardiac toxicity in early breast cancer: addressing the unresolved issues. Circulation. 2012;126(23):2749-63.
7. Tamargo J, Caballero R, Delpón E. Drug-induced atrial fibrillation. Expert Opin Drug Saf. 2012;11(4):615-34.
8. Suter TM, Ewer MS. Cancer drugs and the heart: importance and management. Eur Heart J. 2013;34(15):1102-11.
9. Siu CW, Tung HM, Chu KW, Jim MH, Lau CP, Tse HF. Prevalence and predictors of new-onset atrial fibrillation after elective surgery for colorectal cancer. Pacing Clin Electrophysiol. 2005;28 Suppl 1:S120-3.
10. Riber LP, Christensen TD, Jensen HK, Hoejsgaard A, Pilegaard HK. Amiodarone significantly decreases atrial fibrillation in patients undergoing surgery for lung cancer. Ann Thorac Surg. 2012;94(2):339-44.
11. Nojiri T, Yamamoto K, Maeda H, Takeuchi Y, Funakoshi Y, Inoue M, et al. Effect of low-dose human atrial natriuretic peptide on postoperative atrial fibrillation in patients undergoing pulmonary resection for lung cancer: a double-blind, placebo-controlled study. J Thorac Cardiovasc Surg. 2012;143(2):488-94.
12. Nojiri T, Yamamoto K, Maeda H, Takeuchi Y, Funakoshi Y, Maekura R, et al. Efficacy of low-dose landiolol, an ultrashort-acting β-blocker, on postoperative atrial fibrillation in patients undergoing pulmonary resection for lung cancer. Gen Thorac Cardiovasc Surg. 2011;59(12):799-805.
13. Morelli A, Ertmer C, Westphal M, Rehberg S, Kampmeier T, Ligges S, et al. Effect of heart rate control with esmolol on hemodynamic and clinical outcomes in patients with septic shock: a randomized clinical trial. JAMA. 2013;310(16):1683-91.
14. Kuipers S, Klein Klouwenberg P, Cremer OL. Incidence, risk factors and outcomes of new-onset atrial fibrillation in patients with sepsis: a systematic review. Crit Care. 2014;18(6):688.
15. January CT, Wann LS, Alpert JS, Calkins H, Cigarroa JE, Cleveland JC Jr, et al; American College of Cardiology/American Heart Association Task Force on Practice Guidelines. 2014 AHA/ACC/HRS Guideline for the management of patients with atrial fibrillation: a report of the American College of Cardiology/American Heart Association Task Force on Practice Guidelines and the Heart Rhythm Society. J Am Coll Cardiol. 2014;64(21):e1-76. Erratum in: J Am Coll Cardiol. 2014;64(21):2305-7.
16. Redline S, Yenokyan G, Gottlieb DJ, Shahar E, O'Connor GT, Resnick HE, et al. Obstructive sleep apnea-hypopnea and incident stroke: the sleep heart health study. Am J Respir Crit Care Med. 2010;182(2):269-77.
17. Mansukhani MP, Calvin AD, Kolla BP, Brown RD Jr, Lipford MC, Somers VK, et al. The association between atrial fibrillation and stroke in patients with obstructive sleep apnea: a population-based case-control study. Sleep Med. 2013;14(3):243-6.

18. Abramson JL, Jurkovitz CT, Vaccarino V, Weintraub WS, McClellan W. Chronic kidney disease, anemia, and incident stroke in a middle-aged, community-based population: the ARIC Study. Kidney Int. 2003;64(2):610-5.

19. Xu D, Murakoshi N, Sairenchi T, Irie F, Igarashi M, Nogami A, et al. Anemia and reduced kidney function as risk factors for new onset of atrial fibrillation (from the Ibaraki Prefectural Health Study). Am J Cardiol. 2015;115(3):328-33.

20. Herzog CA, Asinger RW, Berger AK, Charytan DM, Díez J, Hart RG, et al. Cardiovascular disease in chronic kidney disease. A clinical update from Kidney Disease: Improving Global Outcomes (KDIGO). Kidney Int. 2011;80(6):572-86.

21. Guzzetti S, Costantino G, Sada S, Fundarò C. Colorectal cancer and atrial fibrillation: a case-control study. Am J Med. 2002;112(7):587-8.

22. Guzzetti S, Costantino G, Vernocchi A, Sada S, Fundarò C. First diagnosis of colorectal or breast cancer and prevalence of atrial fibrillation. Intern Emerg Med. 2008;3(3):227-31.

23. Hu YF, Liu CJ, Chang PM, Tsao HM, Lin YJ, Chang SL, et al. Incident thromboembolism and heart failure associated with new-onset atrial fibrillation in cancer patients. Int J Cardiol. 2013;165(2):355-7.

24. Erichsen R, Christiansen CF, Mehnert F, Weiss NS, Baron JA, Sørensen HT. Colorectal cancer and risk of atrial fibrillation and flutter: a population-based case-control study. Intern Emerg Med. 2012;7(5):431-8.

25. Dyszkiewicz W, Skrzypczak M. Atrial fibrillation after surgery of the lung : clinical analysis of risk factors. Eur J Cardiothorac Surg. 1998;13(6):625-8.

26. Roselli EE, Murthy SC, Rice TW, Houghtaling PL, Pierce CD, Karchmer DP, et al. Atrial fibrillation complicating lung cancer resection. J Thorac Cardiovasc Surg. 2005;130(2):438-44.

27. Salvatici M, Cardinale D, Spaggiari L, Veglia F, Tedesco CC, Solli P, et al. Atrial fibrillation after thoracic surgery for lung cancer: use of a single cut-off value of N-terminal pro-B type natriuretic peptide to identify patients at risk. Biomarkers. 2010;15(3):259-65.

28. Nojiri T, Maeda H, Takeuchi Y, Funakoshi Y, Maekura R, Yamamoto K, et al. Predictive value of preoperative tissue Doppler echocardiographic analysis for postoperative atrial fibrillation after pulmonary resection for lung cancer. J Thorac Cardiovasc Surg. 2010;140(4):764-8.

29. Imperatori A, Mariscalco G, Riganti G, Rotolo N, Conti V, Dominioni L. Atrial fibrillation after pulmonary lobectomy for lung cancer affects long-term survival in a prospective single-center study. J Cardiothorac Surg. 2012;7:4.

30. Murthy SC, Law S, Whooley BP, Alexandrou A, Chu K-M, Wong J. Atrial fibrillation after esophagectomy is a marker for postoperative morbidity and mortality. J Thorac Cardiovasc Surg. 2003;126(4):1162-7.

31. Onaitis M, D'Amico T, Zhao Y, O'Brien S, Harpole D. Risk factors for atrial fibrillation after lung cancer surgery: analysis of the Society of Thoracic Surgeons general thoracic surgery database. Ann Thorac Surg. 2010;90(2):368-74.

32. Christian SA, Schorr C, Ferchau L, Jarbrink ME, Parrillo JE, Gerber DR. Clinical characteristics and outcomes of septic patients with new-onset atrial fibrillation. J Crit Care. 2008;23(4):532-6.

33. Salman S, Bajwa A, Gajic O, Afessa B. Paroxysmal atrial fibrillation in critically ill patients with sepsis. J Intensive Care Med. 2008;23(3):178-83.

34. Meierhenrich R, Steinhilber E, Eggermann C, Weiss M, Voglic S, Bögelein D, et al. Incidence and prognostic impact of new-onset atrial fibrillation in patients with septic shock: a prospective observational study. Crit Care. 2010;14(3):R108.

35. Lee-Iannotti JK, Capampangan DJ, Hoffman-Snyder C, Wellik KE, Patel B, Tondato F, et al. New-onset atrial fibrillation in severe sepsis and risk of stroke and death: a critically appraised. Neurologist. 2012;18(4):239-43.

36. Walkey AJ, Hammill BG, Curtis LH, Benjamin EJ. Long-term outcomes following development of new-onset atrial fibrillation during sepsis. Chest. 2014;146(5):1187-95.

37. Sidney S, Sorel M, Quesenberry CP Jr, DeLuise C, Lanes S, Eisner MD. COPD and incident cardiovascular disease hospitalizations and mortality: Kaiser Permanente Medical Care Program. Chest. 2005;128(4):2068-75.

38. Buch P, Friberg J, Scharling H, Lange P, Prescott E. Reduced lung function and risk of atrial fibrillation in The Copenhagen City Heart Study. Eur Respir J. 2003;21(6):1012-6.

39. Li J, Agarwal SK, Alonso A, Blecker S, Chamberlain AM, London SJ, et al. Airflow obstruction, lung function, and incidence of atrial fibrillation: the Atherosclerosis Risk in Communities (ARIC) study. Circulation. 2014;129(9):971-80.

40. Steer J, Gibson J, Bourke SC. The DECAF Score: predicting hospital mortality in exacerbations of chronic obstructive pulmonary disease. Thorax. 2012;67(11):970-6.

41. Fuso L, Incalzi RA, Pistelli R, Muzzolon R, Valente S, Pagliari G, et al. Predicting mortality of patients hospitalized for acutely exacerbated chronic obstructive pulmonary disease. Am J Med. 1995;98(3):272-7.

42. Mehra R, Benjamin EJ, Shahar E, Gottlieb DJ, Nawabit R, Kirchner HL, et al. Association of nocturnal arrhythmias with sleep-disordered breathing: The Sleep Heart Health Study. Am J Respir Crit Care Med. 2006;173(8):910-6.

43. Gami AS, Hodge DO, Herges RM, Olson EJ, Nykodym J, Kara T, et al. Obstructive sleep apnea, obesity, and the risk of incident atrial fibrillation. J Am Coll Cardiol. 2007;49(5):565-71.

44. Gami AS, Pressman G, Caples SM, Kanagala R, Gard JJ, Davison DE, et al. Association of atrial fibrillation and obstructive sleep apnea. Circulation. 2004;110(4):364-7.

45. Yaranov DM, Smyrlis A, Usatii N, Butler A, Petrini JR, Mendez J, et al. Effect of obstructive sleep apnea on frequency of stroke in patients with atrial fibrillation. Am J Cardiol. 2015;115(4):461-5.

46. Ananthapanyasut W, Napan S, Rudolph EH, Harindhanavudhi T, Ayash H, Guglielmi KE, et al. Prevalence of atrial fibrillation and its predictors in nondialysis patients with chronic kidney disease. Clin J Am Soc Nephrol. 2010;5(2):173-81.

47. Soliman EZ, Prineas RJ, Go AS, Xie D, Lash JP, Rahman M, et al; Chronic Renal Insufficiency Cohort (CRIC) Study Group. Chronic kidney disease and prevalent atrial fibrillation: the Chronic Renal Insufficiency Cohort (CRIC). Am Heart J. 2010;159(6):1102-7. Erratum in: Am Heart J. 2010;160(6):1190.

48. Bansal N, Fan D, Hsu CY, Ordonez JD, Go AS. Incident atrial fibrillation and risk of death in adults with chronic kidney disease. J Am Heart Assoc. 2014;3(5):e001303.

49. Alonso A, Lopez FL, Matsushita K, Loehr LR, Agarwal SK, Chen LY, et al. Chronic kidney disease is associated with the incidence of atrial fibrillation: the Atherosclerosis Risk in Communities (ARIC) study. Circulation. 2011;123(25):2946-53.

50. Nelson SE, Shroff GR, Li S, Herzog CA. Impact of chronic kidney disease on risk of incident atrial fibrillation and subsequent survival in medicare patients. J Am Heart Assoc. 2012;1(4):e002097.

51. Watanabe H, Watanabe T, Sasaki S, Nagai K, Roden DM, Aizawa Y. Close bidirectional relationship between chronic kidney disease and atrial fibrillation: the Niigata preventive medicine study. Am Heart J. 2009;158(4):629-36.

52. Bansal N, Fan D, Hsu CY, Ordonez JD, Marcus GM, Go AS. Incident atrial fibrillation and risk of end-stage renal disease in adults with chronic kidney disease. Circulation. 2013;127(5):569-74.

53. Providência R, Marijon E, Boveda S, Barra S, Narayanan K, Le Heuzey JY, et al. Meta-analysis of the influence of chronic kidney disease on the risk of thromboembolism among patients with nonvalvular atrial fibrillation. Am J Cardiol. 2014;114(4):646-53.
